# Direct and indirect measurement of somatic cell count as indicator of intramammary infection in dairy goats

**DOI:** 10.1186/1751-0147-53-15

**Published:** 2011-03-04

**Authors:** Ylva Persson, Ida Olofsson

**Affiliations:** 1 Department of Animal Health and Antimicrobial Strategies, National Veterinary Institute/Swedish Dairy Association, Uppsala, Sweden; 2 Dvärsätt 342, 83541 Dvärsätt, Sweden

## Abstract

**Background:**

Mastitis is the most important and costly disease in dairy goat production. Subclinical mastitis is common in goats and is mainly caused by contagious bacteria. Several methods to diagnose subclinical mastitis are available. In this study indirect measurement of somatic cell count (SCC) by California Mastitis Test (CMT) and direct measurement of SCC using a portable deLaval cell counter (DCC) are evaluated. Swedish goat farmers would primarily benefit from diagnostic methods that can be used at the farm. The purpose of the study was to evaluate SCC measured by CMT and DCC as possible markers for intramammary infection (IMI) in goats without clinical symptoms of mastitis. Moreover to see how well indirect measurement of SCC (CMT) corresponded to direct measurement of SCC (DCC).

**Method:**

Udder half milk samples were collected once from dairy goats (n = 111), in five different farms in Northern and Central Sweden. Only clinically healthy animals were included in the study. All goats were in mid to late lactation at sampling. Milk samples were analyzed for SCC by CMT and DCC at the farm, and for bacterial growth at the laboratory.

**Results:**

Intramammary infection, defined as growth of udder pathogens, was found in 39 (18%) of the milk samples. No growth was found in 180 (81%) samples while 3 (1%) samples were contaminated. The most frequently isolated bacterial species was coagulase negative staphylococci (CNS) (72% of all isolates), followed by *Staphylococcus aureus *(23% of all isolates). Somatic cell count measured by DCC was strongly (p = 0.000) associated with bacterial growth. There was also a very strong association between CMT and bacterial growth. CMT 1 was associated with freedom of IMI while CMT ≥2 was associated with IMI. Indirect measurement of SCC by CMT was well correlated with SCC measured by DCC.

**Conclusions:**

According to the results, SCC measured with CMT or DCC can predict udder infection in goats, and CMT can be used as a predictor of the SCC.

## Background

Mastitis is the most important and costly disease in dairy goat production in the Nordic countries (Indrebö unpubl. 1987) and therefore important to diagnose and control. While clinical mastitis is rather easy to detect, animals with subclinical mastitis are often difficult to find since there is a lack of reliable diagnostic methods; especially at farm level [1]. Subclinical mastitis is an important disease since it can lead to reduced milk production, decreased milk quality for dairy purposes and poor milk hygiene; especially important when unpasteurized milk is used for cheese production.

Subclinical mastitis in goats is common [2] and is mainly caused by bacteria; coagulase negative staphylococci (CNS) and *Staphylococcus aureus *(*S. aureus*) being the most common pathogens ([3], [4], Mörk et al., unpubl. 2007). Undiagnosed subclinical mastitis might lead to poor herd udder health due to shedding of udder pathogens from subclinical intramammary infections (IMI). Presence of IMI may be diagnosed indirectly by measuring markers of inflammation in milk. The most important marker is somatic cell count (SCC), which can be measured by both indirect and direct methods. Swedish goat farmers would primarily benefit from diagnostic methods that can be used at the farm, since goat production in Sweden is of a fairly low scale.

Somatic cell count is the most widely used indicator of udder health in cow, sheep and goat milk, but unfortunately SCC is difficult to interpret in goats. Compared to sheep and cows, SCC in goat milk is relatively high also in the healthy udder and it increases throughout the lactation and also with parity [5]. There is also a great variation in SCC among farms and among individuals [6]. However, elevated SCC is, according to Poutrel *et al. *[7], mainly a response to infection. Therefore, measurement of SCC seems likely to be a reliable way to detect goats with IMI. In goats, the milk SCC is more influenced by normal physiological factors than in cows. Therefore, standards for SCC in milk established for cows are not appropriate for goats. Though, to be able to eliminate and prevent goat IMI by using SCC in milk, there is a need for standards and guidelines appropriate for goats. A reliable direct method of measuring SCC is by using an automatic cell counter; either by using a portable cell counter at the farm, or by sending milk samples to a laboratory for measurement in, for example, a Fossomatic cell counter. The advantage with an automatic cell counter is that it is objective and accurate. Disadvantages are that it can be time consuming if sent to a laboratory or costly as expensive equipment is required when used at the farm.

California Mastitis Test (CMT) is a common indirect method of measuring SCC in cows, but some authors claim that CMT is an unreliable method for diagnosing IMI in goats [4], [6]. Other studies, however, report that CMT may be useful for detection of healthy udders [8,9]. The main advantages with CMT are that it is quick, cheap and simple and that it is an "animal-side" test.

The purpose of the study was to evaluate SCC measured with CMT and a portable automatic cell counter (DeLavals cell counter; DCC[10]) as a possible marker for IMI in goats without clinical signs of mastitis. Another aim was to evaluate how well the CMT and DCC results agreed with each other.

## Methods

### Farms and animals

Dairy goats (n = 111), mainly of the Swedish landrace breed, in five different farms (28-165 goats) in Northern and Central Sweden were sampled once in late summer 2008 by the same person. Four farms were sampled at morning milking and one at evening milking. Only clinically healthy animals without changes in udder consistency or milk appearance were included in the study. All goats were in mid to late lactation at sampling.

### Milk sampling and measurement of SCC

All milk samples were collected immediately before machine milking. Milk were tested by CMT and graded from 1 to 5. The scores are ranked according to an increase in viscosity, where the highest viscosity (CMT 5) is more or less correlated to the highest SCC. An aseptic milk sample was then collected from each udder half and sent to the National Veterinary Institute for bacteriological analysis. Milk from each udder half was also collected in test tubes for further cell counting. Milk aliquots were analyzed at the farm the same day with the DCC (DeLaval International AB, Tumba, Sweden[10]).

### Bacteriological examinations

Bacteriological analysis was performed according to accredited routines at the National Veterinary Institute, Uppsala, Sweden. Milk samples (10 μl) were cultured on blood (5%) agar plates, which were incubated at 37 °C for 16-24 h, and re-evaluated at 48 h. Growth on the plates was confirmed by additional laboratory tests in accordance with the routines at the laboratory. *Staphylococcus aureus (S. aureus) *was identified by means of typical colony morphology, α- and β-hemolysis, or by coagulase reaction (coagulase-positive) when typical hemolysis zones were not present. Coagulase-negative staphylococci (CNS) were identified by typical colony morphology and negative coagulase reaction, but were not further characterized for this paper. Streptococci were determined by colony morphology and CAMP-reaction, and 12 biochemical reactions (hippurate, aesculine, salicine, sorbitol, mannitol, raffinose, lactose, saccharose, inuline, trehalose, starch and glycerine) were used for typing to the species level. A milk sample was classified as positive if at least one colony-forming unit (CFU) of *S. aureus *was isolated. For other agents, the presence of at least three CFU was needed for positive classification. Samples were classified as contaminated if three or more bacterial types were isolated from one milk sample and growth of a major udder pathogen was not identified. If moderate to high growth of a major udder pathogen was found in combination with a few CFU of several contaminating species the sample would be diagnosed as positive for growth of the major udder pathogen. In addition, all isolates of staphylococci were examined for betalactamase production by the ''clover-leaf'' method as described by Bryan and Godfrey [11].

### Statistics

Statistical analyses were performed using R, version 2.7.2 [12].

Cohen's kappa coefficient was used to measure the agreement between CMT and DCC (<0 No agreement, 0-0.2 Slight agreement, 0.2-0.4 Fair agreement, 0.4-0.6 Moderate agreement, 0.6-0.8 Substantial agreement, 0.8-1 Almost perfect agreement).

The performance of CMT and DCC as markers for IMI was evaluated using multiple regression models, with the occurrence of IMI as the dependent variable (yes/no) and the particular marker, as well as age (when appropriate), as covariates. In cases where compensation for correlation within herds or individuals was needed, Generalized Estimating Equations (GEE) were used to estimate the models.

Youden's index [13] was used to optimize the cut-off of the sensitivity and specificity test.

## Results

### Bacteriology and somatic cell count

Intramammary infection, defined as growth of udder pathogens, was found in 39 (18%) milk samples from 30 (27%) goats. No growth was found in 180 (81%) samples while 3 (1%) samples were contaminated. The most frequently isolated bacterial species was CNS followed by *S. aureus*. Of the CNS, 27% was positive for betalactamase production. All *S. aureus *isolates were negative for betalactamase production. Nine goats had IMI in both udder halves and three goats had different types of IMI in the two halves. For more detailed information on bacterial findings, see Table 1. The overall arithmetic mean SCC measured by DCC, was 519 × 10^3 ^cells/ml. Mean SCC of udder halves with bacterial growth or freedom of bacteria were 711 × 10^3 ^cells/ml and 481 × 10^3 ^cells/ml, respectively. The highest SCC (1010 × 10^3^cells/ml) was found in udders positive for *S. aureus*. The percentage of different culture results and corresponding SCC are given in Table 1.

**Table 1:** Culture Results and Corresponding Milk SCC (DCC) and CMT Scores in 222 Udder Halves

Culture results	% of all	% of positive culture	Mean SCC (SD) (1000/ml)	Median CMT
***S. aureus *n = 9**	4	23	1010 (1690)	2

**CNS n = 28**	13	72	651 (700)	2

***S. dysgalactiae *n = 2**	1	5	200 (54)	1

**No growth n = 180**	81		481 (713)	1

**Contaminated sample n = 3**	1		281 (21)	1

**All n = 222**			519 (768)	1

### Comparison between SCC and bacteriology

**Figure 1:**
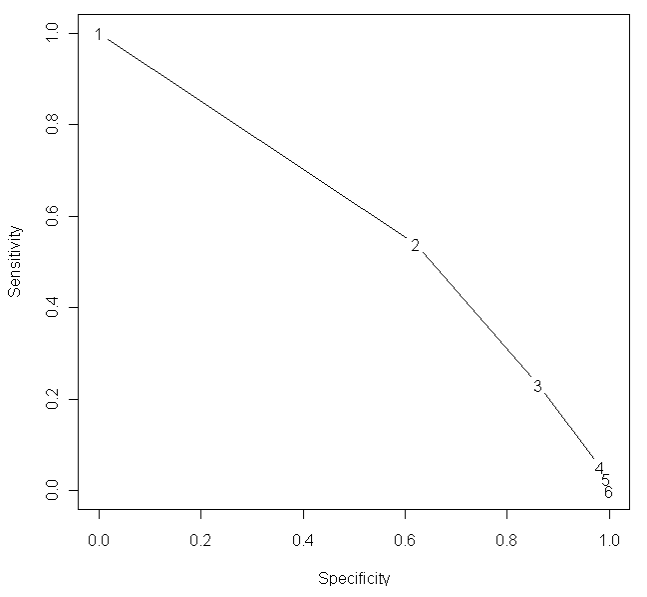
**Sensitivity and specificity of CMT as an indicator of IMI, for different CMT cutoffs**. The graph should be read as follows: If CMT ≥ 2 corresponds to a positive diagnosis and CMT = 1 corresponds to a negative diagnosis; the sensitivity and specificity is 0.54 and 0.62 respectively.

**Figure 2:**
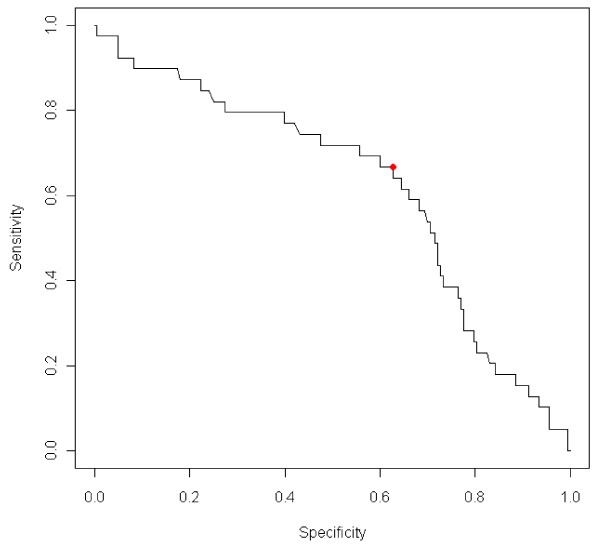
**Sensitivity and specificity for SCC-DCC as an indicator of IMI**. The cutoff in SCC for which Youden's index is maximized (345 × 10^3 ^cells/ml) is highlighted. For that cutoff, the sensitivity is 0.67 and the specificity is 0.63.

Somatic cell counts measured with CMT and DCC were both significantly associated (p = 0.000 and p = 0.01 respectively) with IMI. CMT 1 was associated with freedom of IMI and CMT ≥2 was associated with IMI. Data on IMI at different CMT scores are shown in Table 2. Figure 1 shows the sensitivity and specificity of CMT as an indicator of IMI, for different CMT cut-offs. Figure 2 shows the sensitivity and specificity for SCC measured by DCC (SCC-DCC) as an indicator of IMI.

**Table 2:** Number of samples with bacterial and no growth at different CMT scores

Growth of bacteria	CMT 1	CMT 2	CMT 3	CMT 4	CMT 5
Yes	18	12	7	1	1

No	111	44	22	2	1

### Comparison of SCC measured with CMT and DCC

**Figure 3:**
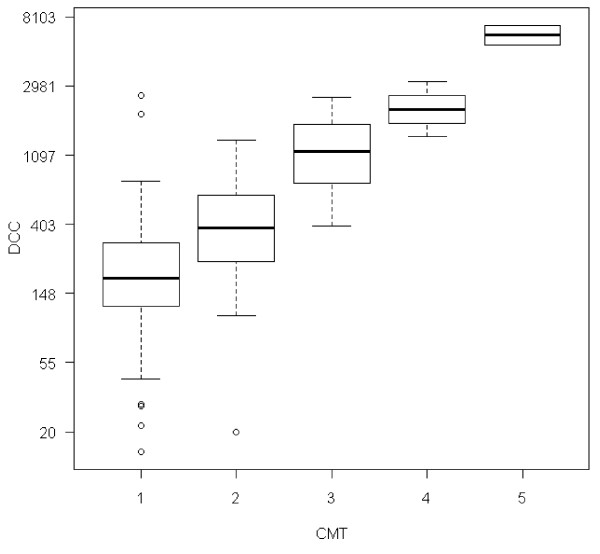
**Correlations between SCC-DCC (×10^3 ^cells/ml) and CMT (1-5)**.

Indirect measurement of SCC by using CMT had a substantial agreement (Cohen's kappa coefficient = 0.64) to SCC measured by DCC. Mean SCC-DCC for CMT 1 was 255 × 10^3 ^cells/ml, for CMT 2; 455 × 10^3 ^cells/ml, for CMT 3; 1265 × 10^3 ^cells/ml, for CMT 4; 2249 × 10^3 ^cells/ml and for CMT 5, mean SCC-DCC was 6291 × 10^3 ^cells/ml. See Figure 3 for relationship between CMT and SCC.

## Discussion

In this study, SCC could predict IMI in goats, measured with indirect (CMT) or direct (DCC) methods.

Only 18% of all udder halves had IMI in the present study. This is lower than in other studies, where the proportion of udder halves with subclinical IMI in goats ranged from 23 to 70% [1,14]. The lower proportion of IMI in this study might be the result of good udder health in the sampled herds. It could also be due to false negative bacterial findings as the goats were only sampled once in this study. For more accurate results, sampling should be repeated at two or more occasions. In general, Swedish goats have a good health status with few problems with infectious diseases. Sweden also has rather small herds which, to some extent, could explain the good udder health. High stocking density, particularly in intensively managed herds, may be associated with large concentrations of microorganisms [4]. In this study, the main pathogen group in infected udder halves was CNS. This is in agreement with other studies on subclinical mastitis in goats [15], [1,4,16].

The mean SCC, measured by DCC, of uninfected udder halves was 478 × 10^3 ^cells/ml. Other authors have reported both lower and higher SCC in goats without IMI [5], [17]. In future studies, it would be interesting to measure SCC throughout the lactation, since SCC in goats can differ markedly between early and late lactation. Goats infected with *S. aureus *had the highest SCC, which is in line with other studies [18], [19].

Somatic cell count measured by CMT agreed with SCC measured with DCC, which is in agreement with other studies [20], [8], [9]. It was also concluded that CMT could predict IMI better than at random, which is in line with a recent study [14]. Goat farmers would therefore benefit from using CMT in their daily work at the farm. CMT is an easy and cheap method, which can be performed as a "goat-side" test. In larger herds, DCC may be a good, but more expensive, alternative for more objective measures of SCC.

## Conclusions

According to these results, SCC measured by CMT or DCC can predict IMI of goats. Moreover, CMT is a good predictor of SCC. Thus, goat farmers can be recommended to use CMT as a "goat-side" test in order to find IMI in goats with no clinical symptoms of mastitis.

## Competing interests

The authors declare that they have no competing interests.

## Authors' contributions

YP conceived of the study and was responsible for its coordination, participated in its design and drafted the manuscript. IO carried out some of the analysis of the study, participated in its design and helped to draft the manuscript. All authors read and approved the final manuscript.
